# Left Atrial Appendage Occlusion Complicated by Appendage Perforation Rescued by Device Deployment

**DOI:** 10.1177/2324709618800108

**Published:** 2018-09-11

**Authors:** Anna Sarcon, Dipayon Roy, David Laughrun, Mary Huntsinger, Jacqueline Schwartz, Jina Sohn, Rahul N. Doshi

**Affiliations:** 1 University of Southern California, Los Angeles, CA, USA

**Keywords:** left atrial appendage occlusion, Watchman, pericardial effusion

## Abstract

The Watchman device is a transcatheter left atrial appendage (LAA) occluding device used in patients with nonvalvular atrial fibrillation (NVAF) and a high CHADS2-VA2SC score who are poor long-term anticoagulation candidates. Pericardial effusion related to device deployment and perforation can be a life-threatening complication. While not common in hands of experienced operators, management may require surgical intervention. Here we present a rare case of LAA perforation, which was corrected by successful repositioning of the device foregoing the need for surgical management.

## Background

The Watchman device is the first US Food and Drug Administration–approved transcatheter left atrial appendage (LAA) occluding device used in patients with nonvalvular atrial fibrillation (NVAF) and a high CHADS_2_-VA_2_SC score who are poor long-term anticoagulation candidates. In NVAF, LAA is the most common source of cardiac thromboembolism,^1^ and its occlusion can reduce the risk of thromboembolic events in a selected patient population.^2^ One of the procedural complications of the Watchman procedure is perforation related to device deployment. In the PROTECT AF trial,^2^ device-related complications were noted to be 8.7%. The specific rate of pericardial effusion was 4.5%, with 3.3% requiring pericardiocentesis or surgical intervention.^3^ More recently, data from the EWOLUTION registry note the rate of pericardial effusion to be 4.1%, with 1.4% of patients requiring surgical management of procedure-related pericardial effusion, with no patients dying as a result of these effusions or subsequent management.^4^ Despite the declining rate of complications with increasing implanter experience and device approval,^3^ a small risk of pericardial effusion requiring surgical intervention remains.^4^ Here we present a rare case of LAA perforation, which was corrected by successful repositioning of the device foregoing the need for surgical management.

## Case Presentation

**Figure 1.**
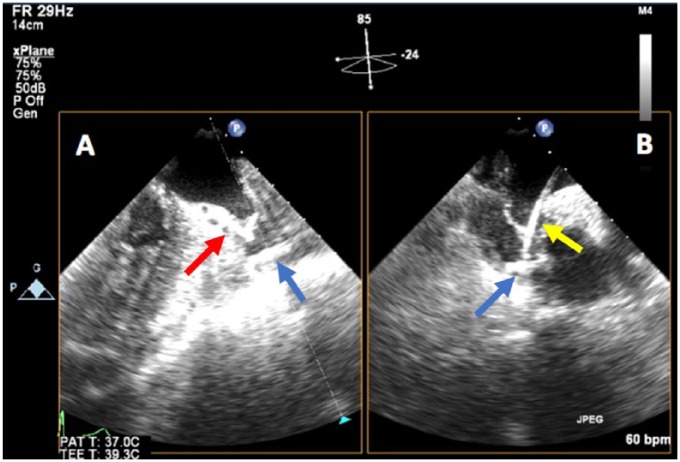
Transesophageal echocardiography images showing the Watchman device located in the patient’s transverse sinus. (A) Crown of the Watchman device (blue arrow) can be seen in the transverse sinus beyond the wall of the left atrial appendage (LAA; red arrow). (B) The sheath (yellow arrow) was not in the plane of view in image “A” but can be seen passing through the LAA into the transverse sinus in image “B.” The Watchman device (blue arrow) remains connected to the delivery system. Transverse sinus is showing evidence of fluid accumulation relative to preprocedure images (not shown).

**Figure 2.**
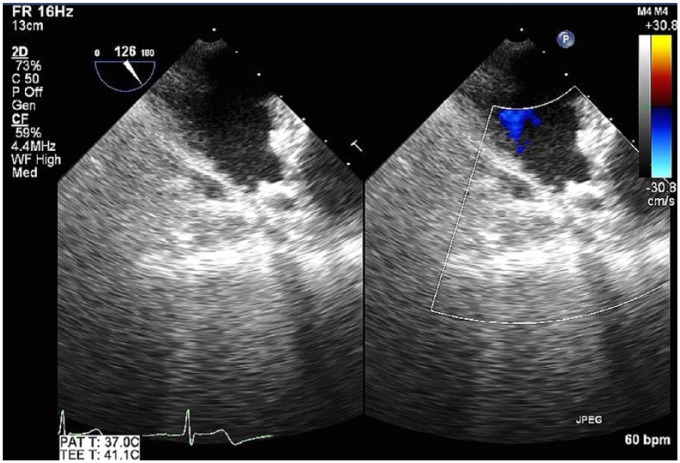
Transesophageal echocardiography images with color Doppler 2 days after device placement shows Watchman deep in the left atrial appendage, without significant peri-device leak. Fluid is no longer apparent in the transverse sinus.

**Figure 3.**
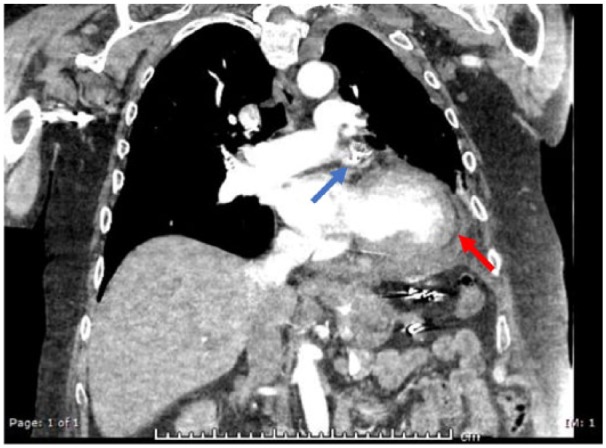
Computed tomography chest 1-month postprocedure showing adequate device positioning (blue arrow) with a small residual pericardial effusion (red arrow).

A 73-year-old man with history of NVAF, hypertension, bradycardia requiring pacemaker implantation, and history of upper gastrointestinal bleed while on anticoagulation was deemed a good candidate for LAA occlusion device implantation. His CHA_2_DS_2_-VASc score was 3, for diastolic heart failure, age, and history of hypertension, and HAS-BLED score was calculated to be 4, putting him at elevated risk for another serious bleed while on therapeutic anticoagulation. The procedure was performed under general anesthesia, and transseptal access was performed with transesophageal echocardiography (TEE) and fluoroscopy guidance. His maximum LAA width measured by echocardiography was 21 mm with maximum depth, 27 mm, measured to the anterior lobe. A 27-mm Watchman device was selected and prepped in the usual fashion and delivered via a dual curve sheath. The activated clotting time during deployment was 213 seconds. As the device was being delivered through the sheath at the area of greatest curvature, the sheath whipped anteriorly before the device exited. Contrast injection during fluoroscopy revealed pericardial staining. Given a presumptive diagnosis of LAA perforation, the device was deployed with sheath remaining in the same distal position given the concern for losing LAA access. After deployment, angiography through the sheath confirmed LAA laceration, with TEE visualization of the device in the transverse sinus (Figure 1). Given increasing hypotension, 2 pericardial drains were placed with acute evacuation of approximately 1 L of blood. The patient was immediately transfused with packed red blood cells, and anticoagulation was reversed with protamine with the sheath remaining in the atrium. The patient was hemodynamically stabilized but required continuous pericardial drainage despite reversal of anticoagulation. Given the presence of an intact proximal portion of the LAA, the decision was made to deploy the device in the LAA to provide an impediment to blood loss. Following device deployment guided primarily by TEE visualization of the delivery sheath, there was an almost immediate cessation of fluid accumulation in the pericardial space. The patient remained hemodynamically stable and transferred to intensive care unit for further monitoring. The patient was kept sedated and intubated to assure stabilization. On postoperative day (POD) 2, a TEE revealed trace pericardial effusion with adequate device position and no peri-device leak (Figure 2). The patient was subsequently extubated. A repeat echocardiogram showed only trivial pericardial effusion on POD 4. The patient was maintained on dual antiplatelet therapy (DAPT) and colchicine, and he was discharged home on POD 5. Computed tomography of the chest, 1-month postimplantation, showed adequate positioning of the device without any evidence of extravasation or device-related thrombus (Figure 3).

## Discussion

While transcatheter closure of LAA is less invasive than surgical exclusion of the LAA from the systemic circulation, it remains a complex procedure and can be associated with serious complications. These complications are mainly attributed to techniques necessary in deploying the LAA closure device, including transseptal puncture and manipulation of stiff wires and guide catheters in the left atrium/LAA.^5^ The Watchman device has been shown to have equivalent follow-up results in randomized controlled trials compared with traditional vitamin K antagonist therapy with a low overall complication rate,^2,6^ as well as a potential for long-term mortality benefits secondary to a decreased risk of bleeding.^7^

One of the most serious complications of LAA occlusion is pericardial effusion resulting in tamponade.^6,8^ Data from the PROTECT-AF trial showed rate of pericardial effusion within 7 days of Watchman implantation to be 4.5%, of which 3.3% of patients required pericardiocentesis or surgical intervention.^6^ Other complications include stroke secondary to thrombus or air embolism, device migration or dislodgement, and vascular complications.^2^ Complication rates related to the procedure appear to be decreasing despite an increased number of novel implanters since device commercialization.^3^

In the PROTECT-AF study, the recommended anticoagulation regimen post device deployment was 45 days of warfarin followed by DAPT until 6 months postprocedure. In this patient, we chose to keep the patient only on DAPT therapy. The EWOLUTION trial has recently been presented and demonstrates low risk of device-related thrombosis with only DAPT therapy.^4^ This approach needs to be validated in prospective trials whether equivalent to vitamin K antagonists. The patient presented here has not shown any evidence of device-related thrombosis on computed tomography scan 1 month postprocedure and has no evidence of recurrent bleeding.

To our knowledge, this is the first case demonstrating the use of the Watchman device to stabilize a patient with a life-threatening perforation of the LAA. The device consists of a Nitinol basket with a semipermeable membrane made of polyethylene terephthalate (Dacron). Thus, the 160-µm pore filter should easily allow blood flow through the membrane. We speculate that the activated clotting cascade initiated by the injury was facilitated by the scaffold provided by the membrane that could enable platelet adhesion and aggregation and lead to immediate cessation of bleeding, similar to hemostatic devices used for surgical bleeding. In addition, the fixation mechanism of the device allowed for stable deployment in the proximal portion of the LAA ostium.

## Conclusion

Device- and procedure-related complications can occur with LAA occlusion and may have life-threatening complications such as cardiac tamponade. We describe a case of Watchman device deployment in which the device complication was managed in part by the device itself.
